# The Association of Quantitative Facial Color Features with Cold Pattern in Traditional East Asian Medicine

**DOI:** 10.1155/2017/9284856

**Published:** 2017-10-17

**Authors:** Sujeong Mun, Ilkoo Ahn, Siwoo Lee

**Affiliations:** Mibyeong Research Center, Korea Institute of Oriental Medicine, 1672 Yuseong-daero, Yuseong-gu, Daejeon 305-811, Republic of Korea

## Abstract

**Introduction:**

Facial diagnosis is a major component of the diagnostic method in traditional East Asian medicine. We investigated the association of quantitative facial color features with cold pattern using a fully automated facial color parameterization system.

**Methods:**

The facial color parameters of 64 participants were obtained from digital photographs using an automatic color correction and color parameter calculation system. Cold pattern severity was evaluated using a questionnaire.

**Results:**

The *a*^⁎^ values of the whole face, lower cheek, and chin were negatively associated with cold pattern score (CPS) (whole face: *B* = −1.048, *P* = 0.021; lower cheek: *B* = −0.494, *P* = 0.007; chin: *B* = −0.640, *P* = 0.031), while *b*^⁎^ value of the lower cheek was positively associated with CPS (*B* = 0.234, *P* = 0.019). The *a*^⁎^ values of the whole face were significantly correlated with specific cold pattern symptoms including cold abdomen (partial *ρ* = −0.354, *P* < 0.01) and cold sensation in the body (partial *ρ* = −0.255, *P* < 0.05).

**Conclusions:**

*a* ^⁎^ values of the whole face were negatively associated with CPS, indicating that individuals with increased levels of cold pattern had paler faces. These findings suggest that objective facial diagnosis has utility for pattern identification.

## 1. Introduction

Pattern identification, also known as syndrome differentiation, is an essential component of the diagnostic process in traditional East Asian medicine (TEAM). Pattern identification is principally used to guide medical intervention; recent studies have suggested that identification improves the rate of successful treatment outcome for both traditional and conventional interventions [1, 2]. Among the basic principles of pattern identification, cold pattern is believed to indicate the nature of imbalance in the body. Cold pattern is indicated when an individual has aversion to cold, preference for warmth, pale face, absence of thirst, cold limbs, clear urine in increased volume, and loose stools [3]. Recent studies indicate that cold pattern is common in women and individuals with low body mass index [4, 5]. Cold pattern has been associated with alterations in metabolic rate [4], thyroid function [6, 7], the sympathetic nervous system [5, 8], the renin-angiotensin system [9], and glucose metabolism [5].

The evaluation of facial complexion has been used as a diagnostic method in TEAM and has also been studied outside of the context of TEAM [10]. Facial diagnosis has traditionally relied on subjective observations and is accordingly influenced by the practitioner's experience and personal knowledge. Thus, several studies have sought to develop an objective and reliable computer-assisted system for facial analysis and diagnosis, specifically in patients with hepatitis and diabetes mellitus [10–15]. Associations of quantified facial color with blood test parameters, health status, and TEAM patterns have also been widely investigated [14, 16–19].

Seo et al. [20] suggested that individuals with cold pattern tend to have pale or white facial color in the forehead, cheeks, and nose, as indicated by decreased *a*^*∗*^ components and increased *L*^*∗*^ components of the CIELAB color system, a universal standardized color system designed to approximate human vision [21]. Regions of interest in the study by Seo et al. were selected by three raters with good or excellent intrarater and interrater reliability; however, this region selection method may have been inconsistent across subjects based on the raters' subjective experiences and personal knowledge. In the present work, we investigated the association of quantitative facial color features with cold pattern using a computerized facial analysis system and color chart correction. We used a digital camera to acquire facial images with automated region selection and the extracted color features were transformed into the CIELAB color space. Automated region selection used both landmark detection and skin detection, which is more accurate than ROI determination by skin detection or approximate location information [11, 13]. Then, associations of facial color parameters with cold pattern symptoms were investigated for each facial region. Automated region selection for regions including the forehead, upper/lower cheeks, nose, chin, and lips permits not only improved reproducibility and convenience, but also the ability to include whole representative facial color as a component of the analysis. Our study therefore represents a first attempt to create an objective method for facial diagnosis and pattern identification focusing on cold pattern.

## 2. Methods

### 2.1. Participants

This cross-sectional study was conducted between December 2014 and March 2015. Facial images and cold pattern data were derived from the Korean Medicine Data Center of the Korea Institute of Oriental Medicine [22]. Sixty-five individuals between the ages of 35 and 44 years, with a body mass index between 18.5 and 25 kg/m^2^, and without any severe fatigue and pain, who are not currently receiving treatment for diseases were included in the study (see attachment for detailed inclusion and exclusion criteria in Supplementary Material available online at https://doi.org/10.1155/2017/9284856). The institutional review board of the hospital approved the study protocol (KHNMCIH 2014-09-010) and informed consent was obtained from all participants prior to inclusion in the study. Data from 64 participants were ultimately included in the analysis because one participant had missing data.

### 2.2. Facial Color

**Figure 1:**
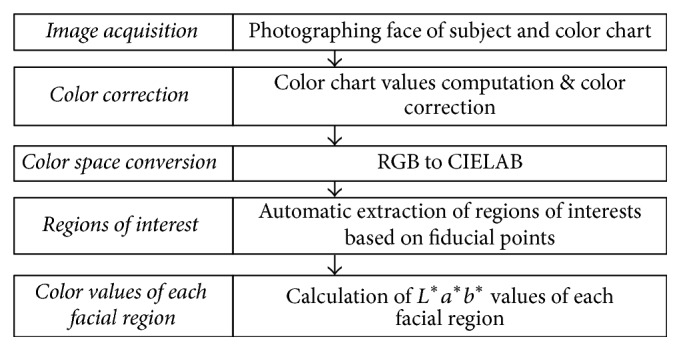
Schematic diagram of facial image analysis.

In this subsection, we describe the calculation method for *L*^*∗*^, *a*^*∗*^, and *b*^*∗*^ values of different facial regions. A schematic diagram of this process is shown in Figure 1.

#### 2.2.1. Image Acquisition

**Figure 2:**
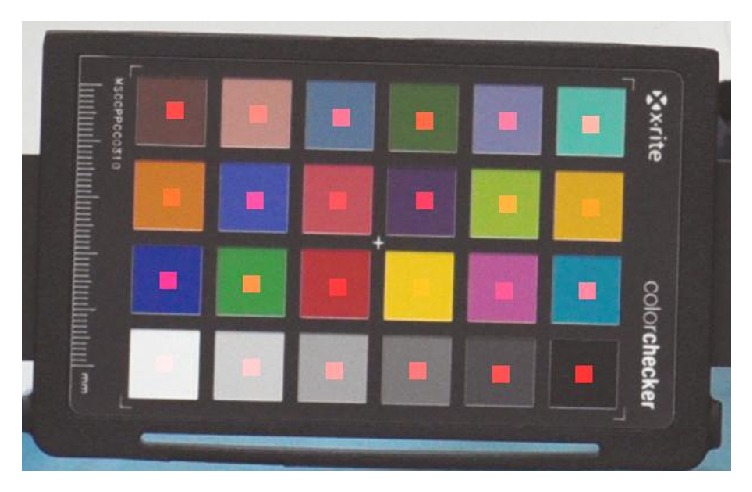
An example image of x-rite color checker passport used in color correction step. The reddish regions of each patch represent the center areas of each patch.

Before facial image acquisition, the purpose and process were explained to participants. Participants were asked to wear a hairband so that their forehead and ears were exposed. Participants were seated comfortably in a chair and the camera was fixed with a tripod at a distance of 1.6 m from the subject. An x-rite color checker passport [23] was placed under each participant's chin as a reference color to correct exposure variability (see Figure 2). Frontal face images were acquired using a digital camera (Nikon D5100 with an 85 mm lens; Nikon Co., Ltd., Japan) equipped with a prime lens (a lens of fixed focal length) by a ruler. To reduce interrater bias, we prepared a strict standard operating procedure (SOP) for photo-taking and observed technicians periodically. If technicians were unable to produce uniform photographs in accordance with the SOP, they reacquired photographs until an appropriate level of accuracy was achieved. Facial images were acquired in the same location and with an outer fluorescent light source and saved at a resolution of 3696 × 2448 pixels in jpeg format.

#### 2.2.2. Image Color Correction

Color correction requires reference color patches be placed in the given image, and then a conversion matrix is used to convert color chart values in the given image to original (target) color chart values. Multiplication of the given image and conversion matrix results in the corrected image. The Munsell color system [23] is commonly used as reference color patches to maintain color consistency across different illumination conditions. In this research, we used an x-rite color checker passport (see Figure 2). The color checker consists of 24 color patches arranged in a 6 × 4 array, with colors selected to represent various natural objects. We used the method of Akkaynak et al. [25] to compute each patch's RGB color values in the color checker. This method automatically detects 24 patches and specifies the center of each patch (center areas are marked by red rectangles in Figure 2). The average RGB values of each patch's central region were calculated and 24 RGB values were obtained.

The following equation was established for the red channel to obtain the conversion matrix:

(1) R1′=α0+α1R1+α2G1+α3B1+α4R1G1+α5G1B1+α6R1B1+α7R12+α8G12+α9B12R2′=α0+α1R2+α2G2+α3B2+α4R2G2+α5G2B2+α6R2B2+α7R22+α8G22+α9B22⋮R24′=α0+α1R24+α2G24+α3B24+α4R24G24+α5G24B24+α6R24B24+α7R242+α8G242+α9B242,

where *R*_1_′,…, *R*_24_′ are the red channel values of the 1st,…, 24th original (golden) patches, respectively, and *R*_1_, *G*_1_, and *B*_1_ are the 1st red, green, and blue channel values of the 1st patch in a given image. Equation (1) can be expressed as matrix (2) as follows:

(2) $$\begin{array}{c} R^{\prime }=CA_{R}\;where\;\;R^{\prime }=\left[\begin{array}{c} R_{1}^{\prime }\\ \vdots \\ R_{24}^{\prime } \end{array}\right],\;\;C=\left[\begin{array}{cccccc} 1 & R_{1} & G_{1} & B_{1} & \cdots & B_{1}^{2}\\ 1 & R_{2} & G_{2} & B_{2} & \cdots & B_{2}^{2}\\ & & & & \ddots & \\ 1 & R_{24} & G_{24} & B_{24} & \cdots & B_{24}^{2} \end{array}\right],\;\;A_{R}=\left[\begin{array}{c} \alpha_{0}\\ \alpha_{1}\\ \vdots \\ \alpha_{9} \end{array}\right]. \end{array}$$

A least squares regression is used to solve for **A**_*R*_. The least squares solution of smallest norm of the linear system is given by

(3) $$\begin{array}{c} {\hat{A}}_{R}=C^{+}R^{\prime }, \end{array}$$

where **C**^+^ is the pseudoinverse of **C**. Now, we have a matrix ${\hat{A}}_{R}$ that converts red, green, and blue pixel values of a given image into a red pixel of a corrected image. This process is applied to other color channels (${\hat{A}}_{G}$ and ${\hat{A}}_{B}$) and least squares solutions of green and blue channels are obtained. Finally, the corrected image is computed by

(4) $$\begin{array}{c} \left[\begin{array}{ccc} \hat{R} & \hat{G} & \hat{B} \end{array}\right]=C\left[\begin{array}{ccc} {\hat{A}}_{R} & {\hat{A}}_{G} & {\hat{A}}_{B} \end{array}\right], \end{array}$$

where $\hat{R}$, $\hat{G}$, and $\hat{B}$ are red, green, and blue pixel values of a corrected image, respectively. The corrected image is then converted from the RGB color space to the CIELAB color space.

#### 2.2.3. Facial Region of Interests (ROI) Extraction

**Figure 3:**
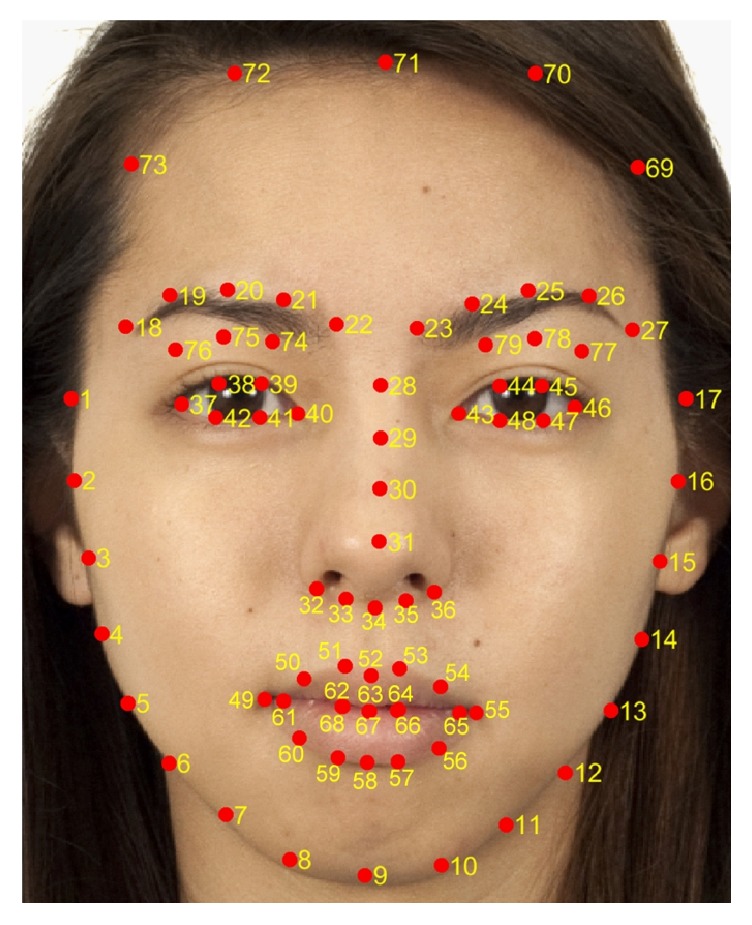
An example of the fiducial points on a frontal face. The original image is from Chicago Face Database [24] which is publicly released image database.

**Figure 4:**
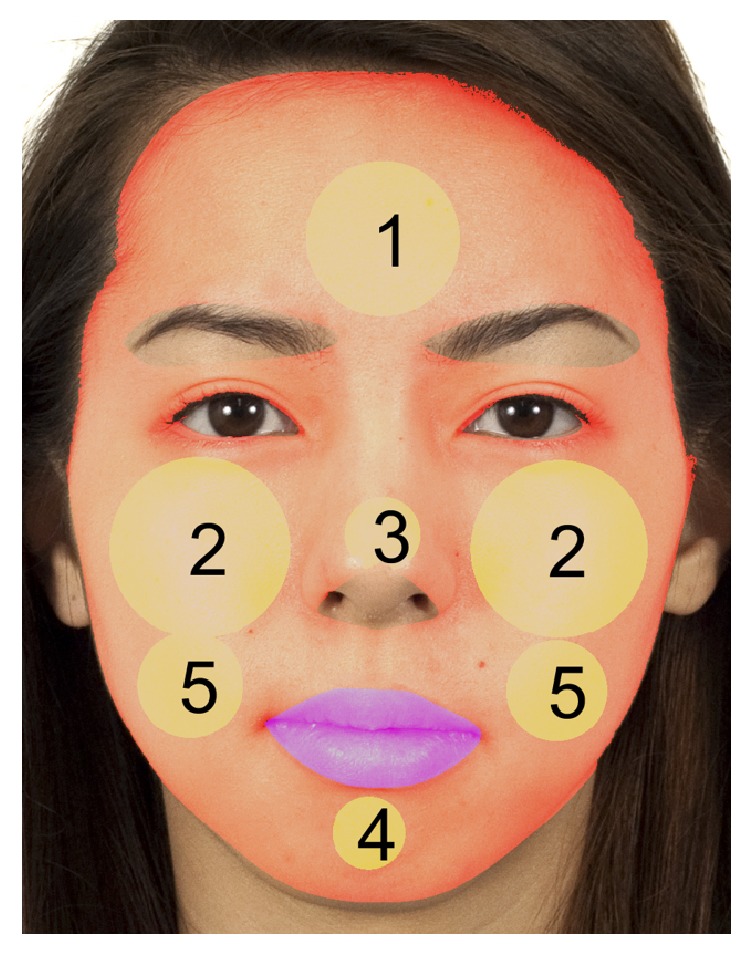
Facial regions of interests (ROI). ROIs (yellow circles) and lip (purple) and whole area of face (reddish). 1: forehead, 2: upper cheek, 3: nose, 4: chin, and 5: lower cheek. The original image is from [24].

To extract the same areas of facial ROIs for all participant face images, we used the facial landmark detector included in the Dlib library [26] which is an implementation of the method of Kazemi and Sullivan [27].  Figure 3 shows an example of the fiducial points on a frontal face (the original image is from Chicago Face Database [24] which is publicly released image database). The number next to the red dot indicates the order of the points in Figure 3. Using these points, we extracted facial ROIs as shown in Figure 4: forehead (1), upper cheek (2), nose (3), chin (4), lower cheek (5), lip (purple), and whole face region (red). The first five facial regions are extracted as a circle and are summarized in Table 1. Taking the nose region as an example, the center point position is determined as the center point of the 30th and 31st fiducial points. The radius of the nose region circle is 1.4 times the distance between the center point and the 31st point.

**Figure 5:**
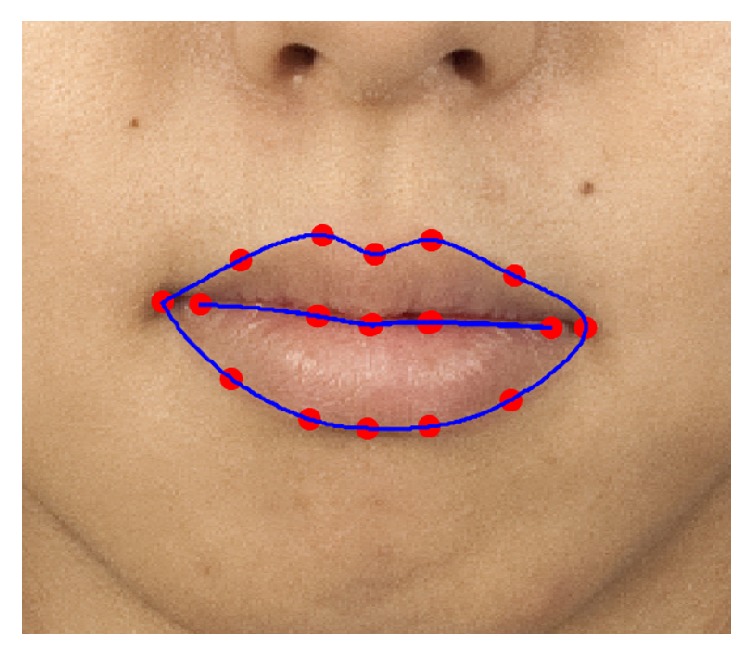
An example of the lip region extraction by spline interpolation. The boundaries of lip region and fiducial points are marked with blue line and red dots, respectively. The original image is from [24].

The lip region is determined by cubic spline data interpolated from points 49 to 60 for the outer boundary and from points 61 to 68 for the inner boundary as shown in Figure 5. The whole face region is decided by spline interpolation from points 1 to 17 and points 69 to 73. Here, the lip, nose tip, eyes, eyebrows, and nonskin regions are excluded from the whole face region. The nose tip region is extracted by spline interpolation from points 31 to 36. The eyes and eyebrows regions are determined in a similar manner. Skin detection is performed by nonparametric histogram-based skin color modeling. We collected a database of skin and nonskin patches from different images from a variety of people under different illumination conditions. Two three-dimensional histogram color models (one for skin and another for nonskin) are constructed in the 24-bit RGB color space. Both histograms (size, 32 bins per color channel) have 32,768 bins each. Each bin of the skin model histogram stores an integer counting the number of times that color value occurs in the entire database of skin images and the same approach is applied to the nonskin model histogram. The counts of each cube histogram are converted into a discrete probability distribution. Finally, an unknown RGB pixel** x** is labeled as skin or nonskin using the Bayesian classifier as follows:

(5) $$\begin{array}{c} x=\left\{\begin{array}{cc} skin, & if\;\;p\left(x\;|\;skin\right)>p\left(x\;|\;\text{nonskin}\right)\\ \text{nonskin,} & otherwise. \end{array}\right. \end{array}$$

After obtaining facial ROIs, each region's trimmed averages of CIELAB *L*^*∗*^, *a*^*∗*^, and *b*^*∗*^ values are computed, where the trimmed average is the mean after discarding the upper and lower quartiles of the distributions. The trimmed mean is a suitable estimator for this study because it excludes the influence of outlying colors in very confined areas such as in rashes, wrinkles, dots, scars, and whiskers in each ROI, instead of reflecting the overall color of the region. The proposed system was implemented in Matlab R2016a (Matlab and Statistics and Machine Learning Toolbox, The MathWorks, Inc., Natick, Massachusetts, United States) in a Win64 environment.

### 2.3. Cold Pattern

The cold pattern questionnaire consisted of eight items that addressed cold pattern symptoms [28], including aversion to cold, preference for heat, cold abdomen, cold hands/feet, cold sensation in the body, pale face, drinking warm water, and clear urine. The questionnaire was self-administered and each question was evaluated on a 4-point scale: 1 = strongly disagree; 2 = disagree; 3 = agree; and 4 = strongly agree. The cold pattern score (CPS) was calculated as the sum of all eight items and ranged from 0 to 32, with higher scores indicating more advanced states of cold pattern. Cronbach's coefficient for this tool was previously reported to be 0.79 [29].

### 2.4. Statistical Analysis

The characteristics of participants are presented as the mean and standard deviation and categorical data are presented as the percentage. To determine the adjusted association of facial colors and cold pattern symptoms, a linear regression analysis was conducted with CPS as a dependent variable and facial color parameters as independent variables, adjusted for sex and age. To assess the association of facial colors and specific cold pattern symptoms, partial correlation coefficients were computed adjusting for sex and age. A *P* value of less than 0.05 was considered to be statistically significant. Statistical analyses were performed using SPSS 22.0 (IBM, Chicago, IL, USA).

## 3. Results 

### 3.1. Characteristics of Participants

The subjects were 32 male participants and 32 female participants with a mean age of 39.2 ± 3.2 years and mean cold pattern score of 20.0 ± 3.6 as shown in Table 2. The mean values of *L*^*∗*^, *a*^*∗*^, and *b*^*∗*^ for each facial region are presented in Table 2. *L*^*∗*^ values were highest in the nose region, *a*^*∗*^ values were highest in the lip region, and *b*^*∗*^ values were highest in the forehead region.

### 3.2. Association of Facial Color with CPS

The results of a multiple regression analysis showed that *a*^*∗*^ value of the whole face was negatively associated with CPS (*B* = −1.048, *P* = 0.021). In a regional analysis, *a*^*∗*^ values of the lower cheek and chin regions were negatively associated with CPS (lower cheek: *B* = −0.494, *P* = 0.007; chin: *B* = −0.640, *P* = 0.031), while *b*^*∗*^ value of the lower cheek was positively associated with CPS (*B* = 0.234, *P* = 0.019) (Table 3).

### 3.3. Correlation of Facial Color with Specific Cold Pattern Symptoms

*L* ^*∗*^ values of the forehead and chin were significantly correlated with cold hands/feet (forehead: partial correlation coefficient = 0.284, *P* < 0.05) and cold sensation in the body (forehead: partial correlation coefficient = 0.292, *P* < 0.05; chin: partial correlation coefficient = 0.258, *P* < 0.05). *a*^*∗*^ values of the whole face were significantly correlated with cold abdomen (partial correlation coefficient = −0.354, *P* < 0.01), cold sensation in the body (partial correlation coefficient = −0.255, *P* < 0.05), and pale face (partial correlation coefficient = −0.349, *P* < 0.01). *a*^*∗*^ values of other regions including the forehead, upper and lower cheek, chin, and lip were significantly correlated with preference for heat, cold abdomen, cold sensation in the body, pale face, and drinking warm water. *b*^*∗*^ value was correlated with aversion to cold only in the lower cheek (partial correlation coefficient = 0.268, *P* < 0.05) (Table 4).

## 4. Discussion

The present study conducted a quantitative assessment of the relationship between facial color features and cold pattern and found that *a*^*∗*^ values of the whole face, lower cheek, and chin were negatively associated with CPS. Moreover, *a*^*∗*^ values of the whole face, forehead, cheek, and chin were negatively associated with cold pattern symptoms including cold abdomen and cold sensation in the body. Among CIELAB color components, *a*^*∗*^ was the most relevant to cold pattern in our cohort and *a*^*∗*^ denotes color on a scale from green (negative values) to red (positive values), such that a negative association between *a*^*∗*^ and CPS is consistent with the TEAM theory that individuals with cold pattern have pale faces [3].

*a* ^*∗*^ values are primarily affected by hemoglobin concentration, blood perfusion to the skin, and blood oxygenation [30, 31]. Although the biological basis of cold pattern remains unclear, cold pattern is often presumed to be related to decreased metabolic rate or diminished function of the whole body [32–34]. It is plausible that a metabolically inactive state demands decreased oxygen in tissue, resulting in decreased skin perfusion and oxygenation and thus decreased *a*^*∗*^ values in the skin. This hypothesis is consistent with a previous study that reported an association between skin *a*^*∗*^ value and physical fitness following repeated bouts of dynamic exercise [35]; in this study, increased *a*^*∗*^ values were assumed to be an adaptive response to demand for increased blood flow to cardiac and skeletal muscle tissues.

We also identified a positive association between *b*^*∗*^ values in the lower cheek area and CPS. *b*^*∗*^ values are mainly affected by melanin and carotenoids [31]; however, it is also reported that *b*^*∗*^ values in skin are negatively associated with hemoglobin concentration [36]. Thus, a relationship of high CPS with decreased *a*^*∗*^ values and increased *b*^*∗*^ values could be explained by decreased cutaneous blood flow or hemoglobin concentrations in individuals with cold pattern, although the specific mechanism of this effect needs to be clarified in future studies.

In our cohort, the colors of the lower cheek and whole face explained the variance of CPS better than the colors of other regions. This is consistent with a previous finding that the colors of the cheek were more indicative of TEAM patterns than other regions (notably, color of the whole face was not included in the analysis) [20]. The cheek is a facial region with high cutaneous blood flow [37]. Blood vessels in the cheek are wider in diameter and located closer to the skin surface than blood vessels in other regions of the face [38]. These traits contribute to the easy visibility of blood perfusion and oxygenation in the cheek. Yet, the reason for less of an association between color of the upper cheek and cold pattern is unclear. The upper cheek is more exposed to sunlight than the lower cheek, such that the influence of sun exposure and increased melanin or freckling may have interfered with the results. The reflection of light on the upper cheek when photos were taken may have also affected the results given the use of a fluorescent light on the ceiling as a light source.

Cold pattern is traditionally diagnosed with comprehensive patient screening and the presence of multiple symptoms rather than a single symptom. In this study, correlations of color parameters with specific cold pattern symptoms were analyzed to verify that the color parameters related to multiple component symptoms of cold pattern and not just the item related to facial color (i.e., self-determined level of paleness of the face). The results showed that *a*^*∗*^ of the whole face had a weak but significant negative correlation with the severity of cold sensation in the abdomen and cold sensation in the body. Interestingly but not surprisingly, correlations were weak between self-determined level of paleness of the face and color parameters, emphasizing the importance of objectivity in facial diagnosis.

The development of an objective pattern identification process has long been desired in order to standardize the diagnostic process of TEAM. Recent studies have shown that various objective variables have significant associations with cold pattern including anthropometric measures such as body mass index [39], resting metabolic rate [4], heart rate variability parameters [8], adiponectin [40], norepinephrine, and changes in glucose and insulin during oral glucose tolerance testing [5]. Together with these previous findings, we hope that our results contribute to the formation of an objective pattern identification system and understanding the underlying mechanism of cold pattern.

Several study limitations should be considered when interpreting our findings. First, we used a subjective self-report cold pattern questionnaire consisting of representative symptoms of cold pattern that were perceived by participants, whereas the traditional diagnosis of a pattern includes a comprehensive inspection (e.g., listening, smelling), inquiry, and palpation of the patient by practitioner (i.e., “the four diagnostic methods”). We selected this questionnaire instead of practitioner-based diagnosis because of the widespread use of structured questionnaires for assessing TEAM patterns in research and the established validity and reliability of the questionnaire used in our study [20, 41, 42]. Additionally, the four diagnostic methods used by a practitioner might lead to different diagnoses for the same individual, resulting in considerable variability across practitioners [43, 44]. Second, although we considered sex and age as covariates, several external factors potentially affecting skin pigmentation were not considered in our analysis. Futures studies should consider the level of sun exposure, which is the most significant external factor affecting skin pigmentation. Third, this study was conducted at a single center with a small sample size. To improve the generalizability of our results, an analysis should be conducted with a larger sample and more strict control of the experimental environment (e.g., light source during photography). Fourth, gloss and luster of the face were not analyzed in this study. In addition to coloring, gloss and luster are important features for facial complexion diagnosis. Thus, future work should assess relationships between facial gloss parameters and specific cold pattern symptoms. Finally, since the results of this study indicate significant associations between color parameters and cold pattern, it will be useful to create a classification model using machine learning techniques for cold pattern identification in the future.

## 5. Conclusions

We investigated the relationship between facial color features and cold pattern using a digital camera and a fully automated facial color parameterization system. *a*^*∗*^ values of the whole face, lower cheek, and chin were negatively associated with cold pattern severity. These results suggest that facial diagnosis can be performed objectively for pattern identification.

## Supplementary Material

This Supplementary Material has 5 Inclusion Criteria and 9 Exclusion Criteria.

## Figures and Tables

**Table 1:** The extraction method of facial regions of interests.

Region (region number)	Description	
	Center point position	Radius
Whole face	Determined by spline interpolation from points 1 to 17 and from 69 to 73. Lip, nose tip (31~36), eyes (43~48, 37~40), eyebrows (23~27 & 77~79, 18~22 & 74~76) regions and nonskin regions are excluded	

Forehead (1)	(pt20 + pt25)/2 + (pt28 − pt63)*∗*0.2	Distance (center point, pt22) *∗* 0.7

Upper cheek (2)	Left: pt31*∗*0.4 + (pt15 + pt16)*∗*0.3 Right: pt31*∗*0.4 + (pt2 + pt3)*∗*0.3	Distance (center point, pt47) *∗* 0.8 Distance (center point, pt42) *∗* 0.8

Lower cheek (5)	Left: pt31*∗*0.25 + pt13*∗*0.75 Right: pt31*∗*0.25 + pt5*∗*0.75	Distance (center point, pt13) *∗* 0.7 Distance (center point, pt5) *∗* 0.7

Nose (3)	(pt30 + pt31)/2	Distance (center point, pt31) *∗* 1.4

Chin (4)	pt9*∗*0.4 + pt58*∗*0.6	Distance (center point, pt58) *∗* 0.8

Lip	Determined by spline interpolation from points 49 to point 60 (outer) and from 61 to 68 (inner)	

**Table 2:** Characteristics of participants.

Number of participants	64	Male	Female	*P* value
		32 (50.0%)	32 (50.0%)	
Age (yr)	39.2 ± 3.2	38.8 ± 3.2	39.7 ± 3.2	0.2588
CPS	20.0 ± 3.6	18.9 ± 3.3	21.1 ± 3.5	**0.0126**
*L* ^*∗*^ (a.u.)				
Whole face	65.5 ± 3.2	64.4 ± 3.8	66.5 ± 2.0	**0.0085 **
Forehead	73.3 ± 3.8	74.1 ± 4.3	72.5 ± 3.3	0.1053
Upper cheek	66.0 ± 3.5	129.1 ± 7.6	135.1 ± 4.7	**0.0004 **
Lower cheek	60.7 ± 3.3	119.7 ± 7.8	123.0 ± 5.0	0.0515
Nose	78.0 ± 3.8	77.5 ± 4.3	78.6 ± 3.3	0.2404
Chin	57.5 ± 5.2	55.2 ± 4.9	59.9 ± 4.5	**0.0002 **
Lip	46.2 ± 3.7	45.6 ± 4.7	46.7 ± 2.5	0.2686
*a* ^*∗*^ (a.u.)				
Whole face	13.0 ± 1.5	13.5 ± 1.6	12.4 ± 1.2	**0.0018 **
Forehead	11.3 ± 1.9	11.6 ± 2.1	11.1 ± 1.6	0.3374
Upper cheek	14.8 ± 1.7	31.1 ± 3.5	28.3 ± 2.8	**0.0008 **
Lower cheek	15.0 ± 1.7	30.4 ± 3.5	29.4 ± 3.0	0.2158
Nose	11.8 ± 2.0	12.4 ± 2.2	11.2 ± 1.6	**0.0134 **
Chin	16.9 ± 2.1	17.0 ± 2.1	16.9 ± 2.2	0.9203
Lip	24.2 ± 2.4	23.7 ± 2.1	24.6 ± 2.6	0.1331
*b* ^*∗*^ (a.u.)				
Whole face	22.2 ± 2.2	21.3 ± 2.2	23.1 ± 1.8	**0.0009 **
Forehead	24.2 ± 2.8	23.3 ± 2.7	25.2 ± 2.6	**0.0041 **
Upper cheek	22.8 ± 2.3	44.3 ± 4.8	46.9 ± 4.2	**0.0242 **
Lower cheek	23.0 ± 2.4	45.0 ± 5.2	47.0 ± 4.3	0.0970
Nose	20.5 ± 2.9	19.6 ± 2.6	21.4 ± 3.0	**0.0131 **
Chin	22.2 ± 3.0	20.9 ± 2.8	23.4 ± 2.6	**0.0004 **
Lip	12.3 ± 1.8	11.9 ± 2.1	12.7 ± 1.3	0.0831

CPS, cold pattern score; *L*^*∗*^, light (+) to dark (−); *a*^*∗*^, red (+) to green (−); *b*^*∗*^, yellow (+) to blue (−). Statistically significant differences between group means as determined by one-way ANOVA are in bold.

**Table 3:** Multiple regression analysis for the association of facial color parameters and cold pattern score.

Region	*L* ^*∗*^		*a* ^*∗*^		*b* ^*∗*^		*R* ^2^	Adjusted *R*^2^
	*B*	*P* value	*B*	*P* value	*B*	*P* value		
Whole	−0.156	0.463	**−1.048**	**0.021**	0.314	0.173	0.244	0.178
Forehead	0.076	0.667	−0.448	0.215	0.205	0.212	0.222	0.153
Upper cheek	−0.029	0.766	−0.370	0.052	0.147	0.153	0.229	0.162
Lower cheek	−0.120	0.213	**−0.494**	**0.007**	**0.234**	**0.019**	0.270	0.206
Nose	−0.007	0.967	−0.264	0.487	0.201	0.256	0.151	0.076
Chin	−0.126	0.374	**−0.640**	**0.031**	0.231	0.191	0.205	0.135
Lip	−0.093	0.527	−0.214	0.262	0.424	0.171	0.142	0.067

Multiple regression analysis was used adjusting for sex and age. Statistically significant results are in bold; *L*^*∗*^, light (+) to dark (−); *a*^*∗*^, red (+) to green (−); *b*^*∗*^, yellow (+) to blue (−); *B*, unstandardized coefficients.

**Table 4:** Partial correlation coefficients between facial color and cold pattern questions.

	Aversion to cold	Preference for heat	Cold abdomen	Cold hands/feet	Cold sensation in body	Pale face	Drink warm water	Clear urine
*L* ^*∗*^								
Whole face	0.018	0.162	0.119	0.147	0.214	0.215	0.121	0.095
Forehead	0.053	0.148	0.194	**0.284** ^*∗*^	**0.292** ^*∗*^	0.089	0.099	0.102
Upper cheek	0.045	0.188	0.103	0.118	0.231	0.166	0.176	0.082
Lower cheek	0.016	0.174	0.137	0.100	0.162	0.223	0.026	0.127
Nose	0.047	0.033	−0.018	0.076	0.182	0.102	0.035	0.108
Chin	0.076	0.138	0.070	0.088	**0.258** ^*∗*^	0.184	0.007	−0.016
Lip	−0.039	0.159	−0.105	−0.129	0.071	0.046	0.099	0.178
*a* ^*∗*^								
Whole face	−0.188	−0.196	**−0.354** ^*∗∗*^	−0.184	**−0.255** ^*∗*^	**−0.349** ^*∗∗*^	−0.048	−0.029
Forehead	−0.164	−0.148	**−0.394** ^*∗∗*^	−0.216	−0.218	−0.185	−0.012	−0.105
Upper cheek	−0.212	**−0.263** ^*∗*^	**−0.291** ^*∗*^	−0.135	−0.243	**−0.289** ^*∗*^	−0.109	0.042
Lower cheek	−0.175	−0.188	**−0.296** ^*∗*^	−0.151	−0.209	**−0.395** ^*∗∗*^	0.003	−0.064
Nose	−0.087	−0.027	−0.132	−0.134	−0.108	−0.21	−0.022	−0.134
Chin	−0.150	−0.138	−0.241	−0.107	**−0.296** ^*∗*^	**−0.411** ^*∗∗*^	0.007	−0.002
Lip	−0.144	−0.082	−0.006	0.128	−0.021	−0.243	**−0.333** ^*∗∗*^	0.073
*b* ^*∗*^								
Whole face	0.176	0.153	0.065	0.049	0.107	0.113	0.125	0.241
Forehead	0.169	0.158	0.052	0.032	0.098	0.112	0.102	0.223
Upper cheek	0.159	0.143	0.019	0.105	0.160	0.146	0.089	0.236
Lower cheek	**0.268** ^*∗*^	0.191	0.076	0.100	0.115	0.067	0.112	0.216
Nose	0.095	0.057	0.060	0.121	0.093	0.175	0.111	0.208
Chin	0.227	0.081	0.123	−0.010	0.034	0.046	0.108	0.144
Lip	0.093	0.233	0.096	−0.037	0.067	0.037	0.111	0.184

Partial correlation coefficients adjusted for sex and age. Statistically significant results are in bold; *L*^*∗*^, light (+) to dark (−); *a*^*∗*^, red (+) to green (−); *b*^*∗*^, yellow (+) to blue (−); ^*∗*^*P* < 0.05; ^*∗∗*^*P* < 0.01.
