# Supplementary material for: The Association of Quantitative Facial Color Features with Cold Pattern in Traditional East Asian Medicine
Source: Evid Based Complement Alternat Med. 2017 Oct 17;2017:9284856. doi: 10.1155/2017/9284856 (PMC5664382; doi:10.1155/2017/9284856)
Supplement: Supplementary file 1 — This Supplementary Material has 5 Inclusion Criteria and 9 Exclusion Criteria. [file 9284856.f1.docx]

The inclusion and exclusion criteria for participants

| Inclusion Criteria |
| --- |
| 1. Healthy men and women aged 35 to 44 years.  2. People whose sleeping hours were between 21:00 and 08:00 in the last week.  3. People who slept for over 7 hours but less than 8 hours during the last week.  4. People who do not have a sleep disorder and who had a PSQI (Pittsburgh Sleep Quality Index) of 5 or less within the past month.  5. People with a BMI greater than 18.5 kg/m^2^ and less than 25 kg/m^2^. |
| Exclusion Criteria |
| 1. People with a Chalder Fatigue Scale score of 19 or higher within the past month.  2. People who have Visual Analog Scale (VAS) score of 40 mm or more within the past month.  3. People who are currently taking medication or receiving treatment for medical, neurological, and psychiatric disorders diagnosed within the last 6 months  4. People who are not fit to undergo metabolic tests due to cardiovascular and respiratory diseases.  5. People who participated in other clinical trials within the past month  6. Among women, pregnant or lactating women, and those menstruating during the test period  7. Smokers and people who quit smoking within the past 3 months  8. People who are not fit to perform a pulse wave test because of a condition such as hand tremor or arrhythmia.  9. People who take medications that can affect clinical outcomes, or those who are deemed unable to follow the clinical guidelines. |
